# Genetic and environmental correlations between subjective wellbeing and experience of life events in adolescence

**DOI:** 10.1007/s00787-017-0997-8

**Published:** 2017-05-16

**Authors:** Robyn E. Wootton, Oliver S. P. Davis, Abigail L. Mottershaw, R. Adele H. Wang, Claire M. A. Haworth

**Affiliations:** 1 School of Experimental Psychology, University of Bristol, Bristol, BS8 1TU UK; 2 MRC Integrative Epidemiology Unit, School of Social and Community Medicine, University of Bristol, Bristol, BS8 2BN UK

**Keywords:** Life events, Subjective wellbeing, Bivariate twin design, Gene–environment correlation

## Abstract

Some life events appear heritable due to the genetic influence on related behaviours. Shared genetic influence between negative behaviours and negative life events has previously been established. This study investigated whether subjective wellbeing and positive life events were genetically associated. Participants in the Twins Early Development Study (aged 16.32 ± .68 years) completed subjective wellbeing and life events assessments via two separate studies (overlapping N for wellbeing and life events measures ranged from 3527 to 9350). We conducted bivariate twin models between both positive and negative life events with subjective wellbeing and related positive psychological traits including subjective happiness, life satisfaction, optimism, hopefulness and gratitude measured at 16 years. Results suggested that the heritability of life events can partially be explained by shared genetic influences with the wellbeing indicators. Wellbeing traits were positively genetically correlated with positive life events and negatively correlated with negative life events (except curiosity where there was no correlation). Those positive traits that drive behaviour (grit and ambition) showed the highest genetic correlation with life events, whereas the reflective trait gratitude was less correlated. This suggests that gene–environment correlations might explain the observed genetic association between life events and wellbeing. Inheriting propensity for positive traits might cause you to seek environments that lead to positive life events and avoid environments which make negative life events more likely.

**Electronic supplementary material:**

The online version of this article (doi:10.1007/s00787-017-0997-8) contains supplementary material, which is available to authorized users.

## Introduction

Environments do not act independently upon an individual; genes shape our environments beyond the way they shape our bodies [1]. Genes influence our environments through their effects on behaviour [2], personality [3] and parenting or socialisation [4]. A meta-analysis of the heritability of environments found an average estimate of 27% [3]. This does not mean that certain genes code for environments. The intermediate step is genetic influence on behaviours and personality traits that guide our experience. This is known as gene–environment correlation [5–8].

Major life events are a measure of our environment with an important influence on our life outcomes. However, their likelihood is also influenced by genetic factors. A meta-analysis revealed life events to be between 24 and 47% heritable with a weighted mean estimate of 28% [3]. Specific life events such as smoking, divorce and diet are all found to have modest to moderate heritability [9–11]. To understand the apparent genetic influence on life events, we need to identify the pathway from genes to experience. Life events are not randomly distributed amongst the population; they are more likely to occur to people in certain environments. People’s behaviour effects the environments they find themselves in. Therefore, identifying intermediate behaviours helps us understand the pathway.

A recent genetically informative study used intermediate behaviours to explore the pathway from genes to negative life events. The intermediate behavioural phenotypes were oppositionality, delinquency, physical aggression, depression, and anxiety [2]. They found that the genetic influences that explained negative life events were very highly correlated with the genetic influences on measured behaviours, for example: .99 genetic correlation with delinquency and .95 with oppositionality. This indicates a gene–environment correlation between the behaviours and negative life events such that genetic predisposition for delinquency and oppositionality makes individuals more likely to encounter environments where negative life events happen to them.

Life events questionnaires tend to contain more negative event items than positive ones because research more frequently focuses on negative mental health outcomes. Furthermore, people may report more negative life events because of societal reluctance to celebrate success. Occurrence of negative life events is associated with risk of depression and negative attributional style [12]. However, attending to positive life events has been shown to increase subjective wellbeing and promote recovery from depression [13]. Therefore, this study considered positive as well as negative life events occurring in adolescence. To avoid assumptions about the experience of these events to young people, their own ratings of valence were used to categorise the events into positive and negative. The period of adolescence is of particular interest because three-quarters of all mental illnesses emerge before a person’s mid 20 s [14]. Therefore, it is critical to understand wellbeing and its effects on the environment before the onset of mental illness if we are to try and prevent it.

Our current study aims to investigate the intermediate behaviours responsible for driving the heritability of positive life events. One such intermediate construct could be subjective wellbeing. Subjective wellbeing is classically defined as life satisfaction and the presence of positive affect in the absence of negative affect [15]. Consequently, measures of life satisfaction and emotional affect or happiness are combined to measure subjective wellbeing [15, 16]. More recently, the focus has broadened to incorporate a range of wellbeing related positive psychological traits including: gratitude, optimism and hopefulness. Here, we consider the links between life events and a diverse range of wellbeing indicators in adolescence.

There is a bidirectional phenotypic association between subjective wellbeing and life events across the lifespan. In adolescence, life events predict both positive and negative affect [16] and positive daily life events predict life satisfaction [16]. Life satisfaction has a prospective effect on the likelihood of marriage, childbirth, divorce, changes in employment [17] and affects the way in which we adapt to significant life events over time [18]. However, these phenotypic studies did not use a genetically sensitive design and, therefore, were unable to conclude whether shared genetic effects drive this relationship. Despite this, there is consistent evidence to suggest a genetic basis for subjective wellbeing, with a meta-analytic estimate of 36% in adult samples [19], and recent evidence of similar levels of heritability in adolescence [20].

Using the twin design, our aim was to understand if heritability of subjective wellbeing could account for the heritability of positive and negative life events. To do this, bivariate twin models were conducted to look for genetic correlations between wellbeing traits and both positive and negative life events. It was hypothesised that there would be a positive genetic correlation between positive life events and wellbeing and a negative genetic correlation for negative life events. We further aimed to measure not just subjective happiness and life satisfaction, but 14 associated positive traits related to wellbeing: subjective happiness, life satisfaction, optimism, curiosity, hopefulness, meaning in life, subjective health, grit, ambition, autonomy, relatedness, competence, trust and gratitude.

## Methods

### Sample

Participants were part of the Twins Early Development Study (TEDS) cohort. TEDS is a sample of twins born in England and Wales between 1994 and 1996 [21]. Twins were excluded from analysis if they were unavailable at first contact, experienced perinatal complications, sex or zygosity was unknown and if medical exclusion criteria were not met. We have no reasons to expect different rates of subjective wellbeing or occurrence of these life events in twins compared with singleton populations.

9336 individuals answered sufficient negative life events items, 9178 individuals answered sufficient positive life events items and 10,915 individuals provided wellbeing data. The number of individuals with overlapping life events and wellbeing data ranges from 3527 to 9350. This variation occurs because measures were collected via two separate studies that included different measures; the first study was an online study, and the second was a postal questionnaire study. The sample is smaller for the online study because funding constraints meant that only twins born in 1994 were invited to take part. The sample is larger for the postal questionnaire study because all TEDS families were invited to take part. The life events measures were included in the larger postal questionnaire study. More details on the method of data collection for each variable are included in Supplementary Table S3. Demographic variables for families responding to online and postal questionnaires are given in Supplementary Table S4 showing that the two samples are comparable. Both were collected at age 16 years, at which point the TEDS sample contained similar rates of parental employment, education, ethnicity, and gender as the overall UK population [22].

Zygosity was determined using a questionnaire [23] completed by the parents at first contact which is 95.7% accurate, as confirmed by DNA data [24]. For those twins who have been genotyped, genetic zygosity is used instead. The five twin categories of sex and zygosity groupings are shown in Supplementary Materials Table S1, indicating the expected proportions of each type of twin in the UK population.

### Life events measures

A reduced version of the Coddington Life Events Scale [25] was used. 20 items of the original 50 were selected for relevance to young adolescents [25]. This was further reduced to 12 items by removing those considered ‘Family Wide’ (occur to both twins simultaneously). For example; hospitalization of a parent is family wide because both twins have the same parents—they either both do, or both do not share this life event. Family wide events cannot be used in twin modelling as the correlation would not differ between MZ and DZ pairs (both should be perfect positive correlation); therefore, only non-family wide life events were used in our analysis.

Life events were self-reported. Each twin indicated whether or not the event had occurred to them in the last 12 months. If the event had occurred, they provided a valence rating from 1 = very unpleasant to 5 = very pleasant. Finally, events were split into positive and negative using the mean valence ratings. Given the scale ranged from 1 to 5, a score of 3 was considered neutral. Anything higher was a positive event and anything lower, a negative event. After excluding the events that were family wide, there were 6 positive and 6 negative events. Individuals who had experienced these events were then given a weighted score calculated as the mean valence minus the neutral score in the case of positive or the neutral score minus the valence in the case of the negative (see Supplementary Table S2 for full list of life events and weights used).

### Measures of wellbeing and associated positive traits

The measures used were: subjective happiness, life satisfaction, optimism, curiosity, hopefulness, meaning in life, subjective health, grit, ambition, autonomy, relatedness, competence, trust and gratitude. Details of the scales used are given in Supplementary Table S3.

### Statistical analysis

#### Twin modelling

 The twin design is genetically informative; it allows us to separate the effect of genes and environments on the variance of a trait. Based on the knowledge that monozygotic (MZ) twins share 100% of their segregating genes and dizygotic twins (DZ) share on average 50%, we can decompose the variance of a trait into A, C and E components. A is additive genetic effects; the extent that genetic differences account for phenotypic differences. C stands for shared environmental factors; environments that affect both twins in the same way. Non-shared environmental factors are represented by E and these are environments that affect the twins differently, making them less similar.

Beyond the variance of one trait, we can also decompose the covariance between traits. This is a bivariate twin model, which is used in this analysis. It allows us to estimate how much of the A, C and E is common between traits. Genetic and environmental covariance was calculated by fitting a bivariate correlated factors solution to each pair of life events and wellbeing traits apart from trust for which a liability threshold model was used. The correlated factors bivariate model is shown in greater detail in Supplementary Figure S1.

All analyses were conducted using the R statistical language [26]. Twin analyses were conducted using the OpenMX package [27] for R and variables were regressed for age and sex before analysis [28].

## Results

### Descriptive statistics

Before weighting by valence, the mean number of positive life events was 1.11 (SD = .92) and negative life events was .51 (SD = .74). The frequencies of life events are shown in Supplementary Table S5.

As shown in Supplementary Table S2, each event was weighted according to the sample mean valence rating for that item. Events were then summed for each individual to create a life events score separately for positive and negative life events. This score incorporated number of events that had occurred to the individual weighted by the mean valence. After weighting, the mean number of positive life events was 1.35 (SD = 1.07) and negative life events was .56 (SD = .80). Due to the positive skew observed for life events scores, each was log transformed before analysis and standardised. Twin correlations were calculated for positive and negative life events comparing each twin with their co-twin. MZ and DZ correlations for positive life events were *r* = .42 and *r* = .31, respectively, and *r* = .28 and *r* = .16 for negative life events (*ps* < .001, *N* = 1527 complete MZ twin pairs and 2666 complete DZ twin pairs for positive and negative life events). Complete twin pairs are those where we have data on both twin 1 and twin 2 in a pair. While the majority of our sample comprises complete twin pairs, the sample also includes some ‘un-paired’ twins, where only one member of the pair has provided data (see Supplementary Table S3 for details of un-paired twins for each measure). Our full information analysis methods allow us to include these un-paired twins, so sample sizes in the tables refer to total number of individuals included. Twin correlations for life events suggested the use of an ACE twin model, due to MZ correlations being less than twice the DZ correlations [29].

### Phenotypic analyses

Pairwise correlations were first conducted between each of the wellbeing measures and both types of life events (Table 1). Correlations with life events were generally weak, being stronger between positive life events than negative life events.

**Table 1:** Phenotypic correlations between each of the 14 wellbeing measures, positive life events and negative life events with 95% confidence intervals

	Positive life events	Negative life events
Subjective happiness	.12 (.10, .14) *N* = 9165	−.10 (−.12, −.08) *N* = 9343
Life satisfaction	.09 (.07, .11) *N* = 9171	−.15 (−.17, −.13) *N* = 9350
Subjective health	.05 (.02, .09) *N* = 3845	−.11 (−.15, −.08) *N* = 3945
Hopefulness	.18 (.15, .22) *N* = 3841	−.10 (−.14, −.07) *N* = 3940
Gratitude	.14 (.10, .17) *N* = 3840	−.09 (−.12, −.06) *N* = 3941
Curiosity	.15 (.11, .18) *N* = 3831	.01 (−.02, .05) *N* = 3932
Grit	.14 (.11, .18) *N* = 3530	−.11 (−.15, −.08) *N* = 3625
Ambition	.20 (.16, .23) *N* = 3527	−.10 (−.14, −.07) *N* = 3622
Optimism	.12 (.08, .15) *N* = 3530	−.12 (−.15, −.09) *N* = 3624
Relatedness	.14 (.12, .16) *N* = 7117	−.06 (−.09, −.04) *N* = 7269
Autonomy	.09 (.07, .12) *N* = 7117	−.10 (−.13, −.08) *N* = 7269
Competence	.22 (.20, .24) *N* = 7114	−.15 (−.17, −.12) *N* = 7267
Meaning in life	.24 (.21, .26) *N* = 7102	−.09 (−.11, −.07) *N* = 7253
Trust	.06 (.03, .10) *N* = 7000	−.16 (−.19, −.13) *N* = 7149

*N* indicates number of individuals with complete data for each wellbeing trait and life events pair. Correlations were calculated using full information maximum likelihood modelling

### Twin modelling

The univariate parameter estimates are given in Table 2. ACE was the best fitting model for positive life events. The nested AE model was not a significantly worse fit for negative life events than an ACE model but ACE was taken forward for bivariate analysis to be consistent with wellbeing parameter estimates.

**Table 2:** Parameter estimates for the best fitting model (with 95% confidence intervals)

	Parameter estimate		
	*a* ^2^ (CI)	*c* ^2^ (CI)	*e* ^2^ (CI)
Positive life events	.23 (.13–.34)	.13 (.05–.21)	.64 (.60–.68)
Negative life events	.33 (.22–.36)	.00 (.00–.08)	.67 (.64–.72)
Subjective happiness	.41 (.36–.44)	.00 (.00–.03)	.59 (.56–.62)
Life satisfaction	.46 (.38–.54)	.10 (.04–.16)	.44 (.42–.47)
Subjective health	.33 (.22–.38)	.00 (.00–.08)	.67 (.62–.72)
Hopefulness	.35 (.21–.40)	.00 (.00–.11)	.65 (.60–.70)
Gratitude	.36 (.22–.45)	.04 (.00–.15)	.60 (.55–.65)
Curiosity	.39 (.32–.44)	.00 (.00–.06)	.61 (.56–.66)
Grit	.38 (.33–.43)	.00 (.00–.10)	.62 (.57–.67)
Ambition	.41 (.32–.45)	.00 (.00–.06)	.59 (.55–.65)
Optimism	.37 (.32–.42)	.00 (.00–.09)	.63 (.58–.68)
Relatedness	.49 (.45–.52)	.00 (.00–.04)	.51 (.48–.55)
Autonomy	.44 (.35–.48)	.00 (.00–.07)	.56 (.52–.59)
Competence	.45 (.39–.49)	.00 (.00–.04)	.55 (.51–.58)
Meaning in life	.46 (.40–.50)	.00 (.00–.04)	.54 (.50–.57)
Trust	.54 (.40–.62)	.00 (.00–.11)	.46 (.38–.54)

**Fig. 1:**
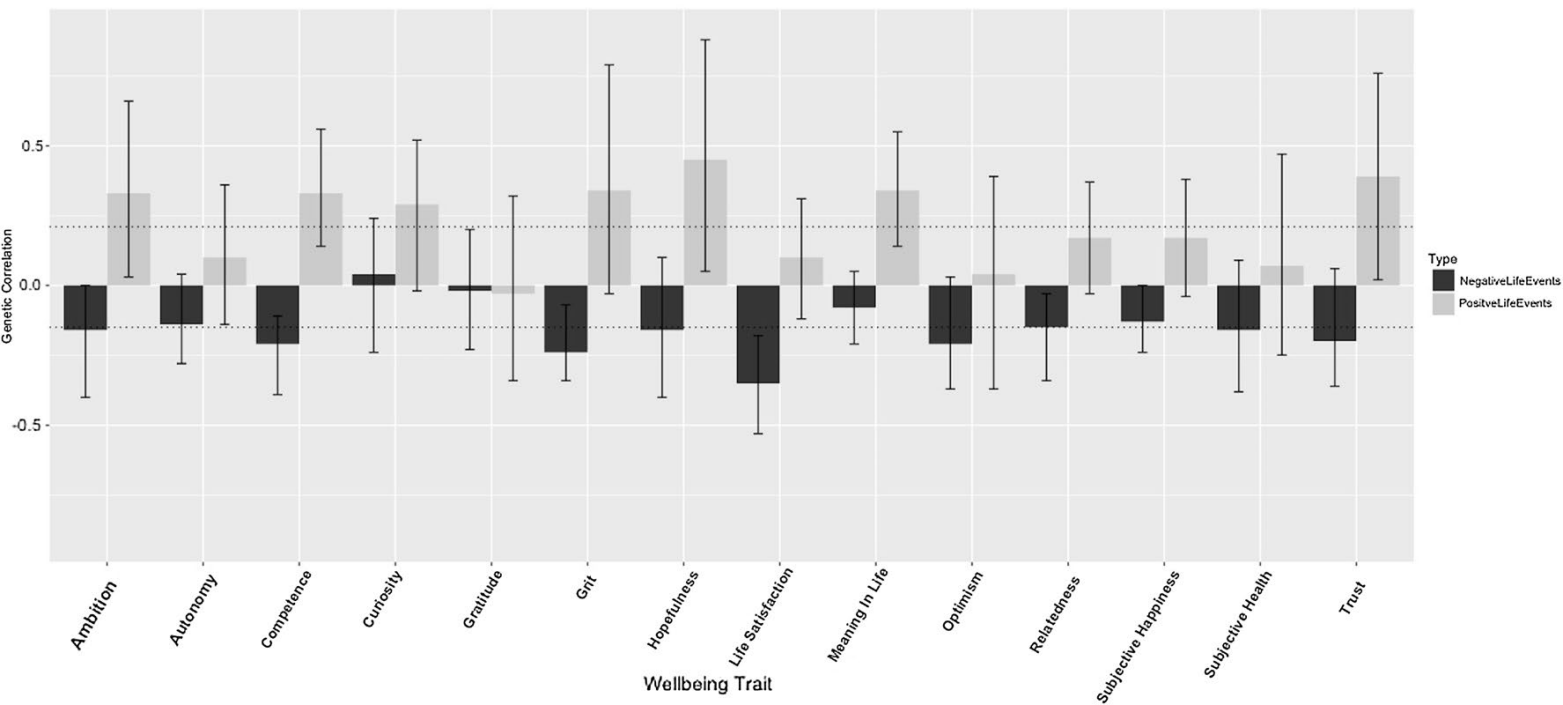
Genetic correlation between positive and negative life events with each of the wellbeing traits (95% CI included)

**Fig. 2:**
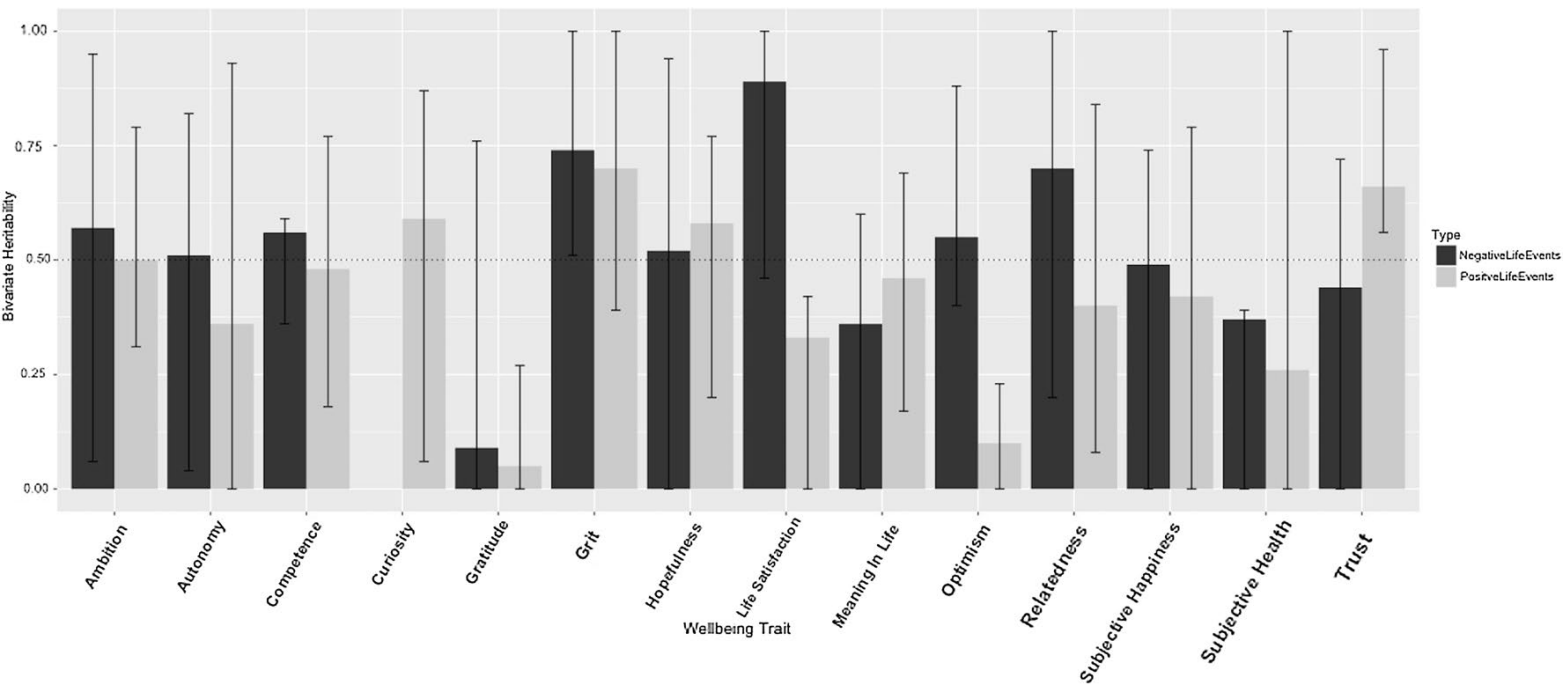
The proportion of the phenotypic correlation explained by shared genetic influence (95% CI included). For phenotypic correlations see Table 1

Bivariate models were run for both positive and negative life events with wellbeing and each of the related positive traits. Initially, Cholesky decompositions were fitted and these converted to Correlated Factors Solutions. The degree of shared genetic influence between life events and wellbeing is represented in Fig. 1 (exact estimates and confidence intervals are included in Supplementary Table S6). The average genetic correlation for the positive life events is .21, and −.15 for negative life events. The average proportion of the phenotypic correlation accounted for by shared genetic effects was .42 for positive life events and .52 for negative life events (See Tables S6 and S7 for further details of all proportions). The estimates for the proportion of the phenotypic correlations explained by genetic factors for each bivariate analysis are presented in Fig. 2. Proportion was not calculated for curiosity and negative life events because there was no significant phenotypic correlation.

**Fig. 3:**
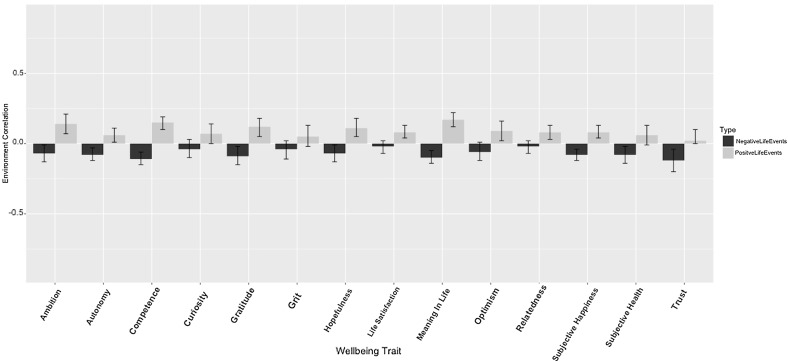
Environmental correlation between positive and negative life events with each of the wellbeing traits (95% CI included)

The estimates of shared E are given in Supplementary Tables S6 and S7 and a graphical representation of these results are given in Fig. 3. The average non-shared environmental correlation for the positive life events is .10, and −.07 for negative life events.

## Discussion

We observed a trend of positive genetic correlation between the wellbeing traits and positive life events. This supports our hypothesis that wellbeing traits are part of the gene–environment correlation that makes life events heritable. Secondly, there was a trend of negative genetic correlation between negative life events and wellbeing. This suggests that the inherited wellbeing traits that drive us towards positive experiences also make us less driven towards negative ones. Roughly, half of the phenotypic correlation was accounted for by environments and half by genetic factors. However, the confidence intervals on these estimates are large due to the small phenotypic correlations.

Small phenotypic correlations between subjective wellbeing and life events result in only low associations being partitioned into genetic and environmental influences. Consequently, the relative shared genetic influence between wellbeing and life events must be interpreted in light of the limited overlap between these measures.

The strength of genetic correlations observed is not as large as seen between negative life events and conduct behaviours or depression and anxiety [2]. McAdams, Gregory and Eley [2] found the highest correlation to be .99 for delinquency and negative life events. However, the negative life events items are quite similar to behaviours we might class as delinquent. For example, suspension from school, being arrested, and being involved in drugs. We would, therefore, expect the correlations to be higher for these more phenotypically similar traits.

In the current study, the average genetic correlation with the wellbeing and related positive measures was .21 for positive life events and −.15 for negative life events. The strength of this effect is small and, therefore, we conclude that heritability of wellbeing traits only partially contributes to heritability of life events. Several confidence intervals overlap zero despite the expected trend being observed. Using a scale with more common life events might make this effect clearer, and indeed a focus on more positive life events would be expected to show a greater overlap with the wellbeing measures. As previously discussed, this effect could be weakened by the fact that less attention is paid to positive life events compared to negative ones. Interventions that direct attention towards positive life events and help people recognise and celebrate their successes could have a dramatic effect on wellbeing.

The average non-shared environmental correlation was minimal (.10 for positive life events and −.07 for negative life events), suggesting that the environments that drive twins to vary for life events are not the same as those that cause variance in wellbeing, despite similar underlying genetic influences on these traits. This is reminiscent of the pattern observed for depression and anxiety; despite a large genetic correlation, they have weak environmental correlations suggesting that environmental experiences steer the genetic propensity to develop into one or the other [30, 31].

Finding a genetic correlation between life events and wellbeing could also indicate the presence of pleiotropy, where the same genes are having different effects on wellbeing compared with life events. This is further suggested by the small phenotypic correlation despite a moderate genetic correlation. Given the heritability and genetic correlations observed we would expect the phenotypic correlations to be higher. In this case, the virtually absent shared environmental influence, and minimal non-shared environmental overlap, is contributing to the lower phenotypic correlation between these measures. We could say, as with depression and anxiety, that although they share a similar genetic propensity, the environments lead to different outcomes (becoming depressed or becoming anxious). While this makes statistical sense, it does not make phenotypic sense in our example. Firstly, wellbeing and life events are not independent outcomes. Secondly, we would not expect genes to act directly on life events; therefore, another mediator is implied. This intermediate behaviour (influenced by similar genes to wellbeing) might be driving the gene–environment correlation with life events. This is supported by a low observed environmental correlation.

As previously discussed, a bidirectional phenotypic association is observed between subjective wellbeing and life events [16–18]. The reasons for this association are reported to be the effect positivity has on creativity, risk-taking and goal-approach behaviour [32]. This explanation fits with another observation on the results: the traits that precede an event (for example: grit and ambition) have a larger genetic correlation than those that are reflective (gratitude). Grit and ambition might be driving the person towards life events whereas gratitude follows behaviour and does not have as large an influence on the events occurring in an individual’s life. Alternatively, it could be that grit and ambition increase our likelihood to appraise events positively rather than driving behaviour. However, if this were the case, we would expect to see a similarly large correlation for gratitude. If goal directed behaviours are driven by positivity to cause positive life events, then it would make sense that the traits associated with goal directed behaviour would share most genetic influence with positive life events.

We cannot rule out the possibility that the correlation between wellbeing and life events is the result of reporting bias: individuals with a positive appraisal bias are more likely to rate their subjective wellbeing as high and more likely to remember positive things happening to them. However, if this were the case we might expect to see higher genetic correlations for positive psychological traits more associated with reflective appraisal, for example, gratitude. The low genetic correlation for gratitude and high genetic correlations for grit and ambition suggest that gene–environment correlation is a more likely explanation.

The fact that the positive psychological traits that drive behaviour are better predictors than more reflective traits suggests a gene–environment correlation. However, there may be other explanations. It could be that the shared genetic influence is affecting wellbeing and life event occurrence independently. It is not necessarily the case that gene–environment correlation explains all the heritability of life events. Genome-wide complex trait analysis (GCTA) has been used to follow up twin analyses that have found heritability estimates for environmental factors, such as education duration [33]. GCTA can also tell us about the heritability of family wide environments, which cannot be examined using a twin design [34]. Also, we know that life events are predictive of wellbeing [16]. The assessed life events could have occurred at any point over the last 12 months and, therefore, could be predicting the wellbeing scores. Some other intermediary factor could be underlying the gene–environment correlation for life events, which in turn affect wellbeing, appearing as a genetic correlation. With the current data, we cannot conclude direction of effect, but hope to have the opportunity to follow up the TEDS twins into young adulthood to assess the longitudinal relationships between life events and wellbeing.

An alternative intermediary factor could be educational attainment. The most common life events were ‘*Outstanding personal achievement*’ and ‘*Failing an important exam*’. Therefore, the underlying mechanism could be that high grit and ambition cause individuals to have higher educational attainment and this is indicated in higher instances of the life event ‘*Outstanding personal achievement*’. Future investigations that include educational attainment in a multivariate model could further explain the gene–environment correlations observed.

The heritability of positive and negative life events has previously been compared using meta-analysis [3]. For the four studies that met inclusion criteria, positive life events were more heritable than negative. This is the opposite pattern found to the current study. This could be explained by the valence of the items being more extreme in the case of the positive events compared with negative. Further, the difference could be due to the fact that the positive events were more frequent than the negative events leaving the negative life events score more zero inflated and therefore less normalised by transformation. This is a common problem of life events but the classic adjustments were made [35]. Another common problem of life events is the reliability of self-report measures. An objective measure of event occurrence was unavailable so report could not be validated. However, twins are only asked to recall life events occurring in the last 12 months rather than in their lifetimes to reduce recall bias.

The adolescent life events selected here are not necessarily generalisable to other age groups. The valence ratings for certain life events (for example: becoming involved with drugs and becoming pregnant) are especially likely to change with age. Further, heritability itself changes with age [36]. Therefore, the assessment of life events at multiple time points and of different types will be an important future consideration [3] as well as longitudinal measures of subjective wellbeing. This will also address the earlier problem that these academic life events might not be representative. It will also enable us to determine direction of effects, for example modelling wellbeing traits at 16 years with life events in early adulthood.

## Conclusion

The current study found shared genetic influence for life events and wellbeing. This furthers a gene–environment correlation explanation of the heritability of positive life events. The research demonstrates that the genetics of life events, wellbeing and related positive traits are outside of the skin as well as under it, genetically driving our behaviours and therefore environments. Genetic correlations are only moderate and phenotypic correlations are weak, indicating that wellbeing is only part of the explanation and further intermediary behaviours and personalities play a role. Other intermediary behaviours could also explain the unexpectedly low observed phenotypic correlation. The fact that wellbeing is the driver of the life events is suggested by the high genetic correlations of grit and ambition and the low genetic correlation of gratitude—a reflective trait. However, direction of effect is only theoretically implied and future consideration of causality is advised.

## Key points

- Gene–environment correlation between negative behaviours and experience of negative life events has previously been established.
- The current study was the first to show a genetic correlation between the experience of positive life events and positive behaviours in adolescence.
- Traits which drive our behaviour (grit and ambition) are more strongly genetically correlated with positive life events than reflective traits (gratitude) which follow behaviours.
- This supports the hypothesis that a genetic propensity towards higher wellbeing causes the young person to seek environments in which positive life events are more likely to occur to them.

## Electronic supplementary material

Below is the link to the electronic supplementary material.

Supplementary material 1 (DOCX 65 kb)
