# Supplementary material for: Genetic and environmental correlations between subjective wellbeing and experience of life events in adolescence
Source: Eur Child Adolesc Psychiatry. 2017 May 16;26(9):1119–27. doi: 10.1007/s00787-017-0997-8 (PMC5591350; doi:10.1007/s00787-017-0997-8)
Supplement: Supplementary file 1 — Supplementary material 1 (DOCX 65 kb) [file 787_2017_997_MOESM1_ESM.docx]

**Genetic and Environmental Correlations between Subjective Wellbeing and Experience of Life Events in Adolescence**

Robyn E Wootton^1,2^*, Oliver S P Davis^2^, Abigail L Mottershaw^1,2^, R. Adele H. Wang^1,2^, Claire M A Haworth^1,2^

1. School of Experimental Psychology, University of Bristol, Bristol, BS8 1TU, UK
2. MRC Integrative Epidemiology Unit, School of Social and Community Medicine, University of Bristol, BS8 2BN, UK

**Index of Supplementary Figures**

1. Correlated factors solution for a bivariate twin model.

**Index of Supplementary Tables**

1. Proportion of sex groups by zygosity.
2. Coddington Life events items administered and scores given to them.
3. Measures of Subjective Wellbeing
4. Representativeness of the TEDS Sample Respondents to Online and Postal Questionnaires
5. Frequencies of the number of life events experienced by individuals.
6. Additive Genetic (A) correlation and Non-shared Environmental (E) correlation from a correlated factors solution between life events and wellbeing.
7. The proportion of the phenotypic correlation explained by bivariate additive genetic variance (A) and bivariate non-shared environmental variance (E) for positive life events and each of the wellbeing traits.
8. The proportion of the phenotypic correlation explained by bivariate additive genetic variance (A) and bivariate non-shared environmental variance (E) for negative life events and each of the wellbeing traits (95% confidence intervals in brackets).

**Supplementary Tables and Figures**

**Trait 1**

**Trait 2**

rA

rC

rE

⎷e_2_

⎷c_2_

⎷a_2_

⎷a_1_

⎷c_1_

⎷e_1_

*Figure S1: Correlated factors solution for a bivariate twin model. This is converted from the mathematically equivalent Cholesky Decomposition but does not require an imposed order of the included variables. rA, rC and rE represent the overlap between A, C and E respectively for trait 1 and trait 2.*

*Table S1: Proportion of sex groups by zygosity.*

|  | N | % |
| --- | --- | --- |
| MZ Male | 1142 | 15 |
| MZ Female | 1640 | 21 |
| DZ Male | 1058 | 14 |
| DZ Female | 1418 | 19 |
| DZ Opposite Sex | 2370 | 31 |

*Note: N is the number individuals with complete life events data.*

*Table S2: Coddington Life events items administered and scores given to them.*

| **Life event** | **Mean Valence** | **Weight** | **Event Type** |
| --- | --- | --- | --- |
| Outstanding personal achievement | 4.50 | 1.50 | Positive |
| Beginning to date | 4.27 | 1.27 | Positive |
| Becoming a member of a church | 4.16 | 1.16 | Positive |
| Moving to a new school or college | 3.85 | .85 | Positive |
| Becoming involved with drugs | 3.33 | .33 | Positive |
| Being sent away from home | 3.27 | .27 | Positive |
| Suspension from school | 2.59 | .41 | Negative |
| Getting pregnant or fathering a pregnancy | 2.21 | .79 | Negative |
| Breaking up with a boyfriend/girlfriend | 1.98 | 1.02 | Negative |
| Being hospitalised for illness or injury | 1.97 | 1.03 | Negative |
| Being responsible for a road accident | 1.74 | 1.26 | Negative |
| Failing an important exam | 1.66 | 1.34 | Negative |

*Table S3: Measures of Subjective Wellbeing*

| Trait | Reference | Chronbach’s α | N Items | Data Collection Type | Total N | N Complete  Pairs | N Unpaired Individuals |
| --- | --- | --- | --- | --- | --- | --- | --- |
| Subjective Happiness | The Subjective Happiness Scale [1] | 0.79 | 4 | Online and Postal | 10574 | 5287 | 325 |
| Life Satisfaction | A composite of the multidimensional student life satisfaction scale [2] and the brief multidimensional student life satisfaction scale. [3] | 0.86 | MSLSS = 21 items;  BMSLSS = 5 items. | Online  Postal | 10576 | 5288 | 323 |
| Meaning in Life | The meaningful life measure [4] | 0.82 | 5 | Postal | 7392 | 3696 | 67 |
| Relatedness | The basic psychological needs satisfaction sale [5] | 0.84 | 8 | Postal | 7424 | 3712 | 52 |
| Autonomy |  | 0.66 | 7 | Postal | 7424 | 3712 | 52 |
| Competence |  | 0.69 | 6 | Postal | 7416 | 3708 | 56 |
| Trust | Single social trust item [6] | - | 1 | Postal |  |  |  |
| Hopefulness | The children’s hope scale [7] | 0.75 | 6 | Online | 4780 | 2390 | 533 |
| Gratitude | The gratitude questionnaire-6 [8] | 0.75 | 6 | Online | 4782 | 2391 | 532 |
| Optimism | The life orientation test – revised [9] | 0.76 | 6 | Online | 4174 | 2087 | 467 |
| Ambition | The ambition scale [10] | 0.75 | 5 | Online | 4170 | 2085 | 469 |
| Curiosity | The curiosity and exploration inventory [11] | 0.74 | 7 | Online | 4764 | 2382 | 538 |
| Grit | The short grit scale [12] | 0.72 | 8 | Online | 4176 | 2088 | 466 |
| Subjective Health | General health single item from the KIDSCREEN-52 generic health-related quality of life questionnaire for children and adolescents [13] | - | 1 | Online | 4796 | 2398 | 525 |

*Table S4: Representativeness of the TEDS Sample Respondents to Online and Postal Questionnaires*

|  | Returned data (*N* families) | % Response rate | % White | % A-levels or higher | % Mother employed | % Father employed | % Female | % MZ |
| --- | --- | --- | --- | --- | --- | --- | --- | --- |
| Online Data Collection | 2996 | 50.6% | 92.4% | 56.4% | 94.6% | 96.7% | 57.8% | 36.9% |
| Postal Data Collection | 4852 | 52.6% | 93.5% | 57.0% | 94.8% | 96.7% | 55.7% | 36.2% |

*Note: % A-levels refers to the mothers’ education qualifications. % Mother and father employed refers to employment status at the time of the year 16 study. MZ = monozygotic twins. All measures are calculated on a family wide basis apart from % female.*

*Table S5: Frequencies of the number of life events experienced by individuals.*

| N Life events | Positive | | Negative | |
| --- | --- | --- | --- | --- |
|  | **N** | **%** | **N** | **%** |
| 0 | 2116 | 28 | 4722 | 62 |
| 1 | 3282 | 43 | 2264 | 30 |
| 2 | 1728 | 23 | 529 | 7 |
| 3 | 431 | 6 | 94 | 1 |
| 4 | 60 | 1 | 11 | <1 |
| 5 | 8 | <1 | 1 | <1 |
| 6 | 3 | <1 | 7 | <1 |

*Table S6: Additive Genetic (A) correlation and Non-shared Environmental (E) correlation from a correlated factors solution between positive and negative life events and each of the wellbeing traits (95% confidence intervals in brackets).*

|  | Positive Life Events | | Negative Life Events | |
| --- | --- | --- | --- | --- |
|  | **Genetic Correlation** | **Environment**  **Correlation** | **Genetic Correlation** | **Environment**  **Correlation** |
| Subjective Happiness | 0.17  (-0.04 , 0.38) | 0.08  (0.04, 0.13) | -0.13  (-0.24, 0.00) | -0.08  (-0.12, -0.04) |
| Life Satisfaction | 0.10  (-0.12, 0.31) | 0.08  (0.04, 0.13) | -0.35  (-0.53, -0.35) | -0.02  (-0.07, 0.02) |
| Subjective Health | 0.07  (-0.25, 0.47) | 0.06  (-0.01, 0.13) | -0.16  (-0.38, 0.09) | -0.08  (-0.14, -0.02) |
| Hopefulness | 0.45  (0.45, 0.88) | 0.11  (0.05, 0.18) | -0.16  (-0.40, 0.10) | -0.07  (-0.13, -0.01) |
| Gratitude | -0.03  (-0.34, 0.32) | 0.12  (0.05, 0.18) | -0.02  (-0.23, 0.20) | -0.09  (-0.15, -0.02) |
| Curiosity | 0.29  (-0.02, 0.52) | 0.07  (0, 0.14) | 0.04  (-0.24, 0.24) | -0.04  (-0.10, 0.03) |
| Grit | 0.34  (0.34, 0.79) | 0.05  (-0.02, 0.13) | -0.24  (-0.34, -0.07) | -0.04  (-0.11, 0.02) |
| Ambition | 0.33  (0.03, 0.66) | 0.14  (0.07, 0.21) | -0.16  (-0.40, 0.00) | -0.07  (-0.13, -0.01) |
| Optimism | 0.04  (-0.37, 0.39) | 0.09  (0.02, 0.16) | -0.21  (-0.37, 0.03) | -0.06  (-0.12, 0.01) |
| Relatedness | 0.17  (-0.03, 0.37) | 0.08  (0.03, 0.13) | -0.15  (-0.34, -0.03) | -0.02  (-0.07, 0.02) |
| Autonomy | 0.10  (0.10, 0.36) | 0.06  (0.01, 0.11) | -0.14  (-0.28, 0.04) | -0.08  (-0.12, -0.03) |
| Competence | 0.33  (0.33, 0.56) | 0.15  (0.1, 0.19) | -0.21  (-0.39, -0.11) | -0.11  (-0.15, -0.06) |
| Meaning in Life | 0.34  (0.34, 0.55) | 0.17  (0.12, 0.22) | -0.08  (-0.21, 0.05) | -0.10  (-0.14, -0.05) |
| Trust | 0.34  (0.03-0.76) | -0.01  (-0.10, 0.08) | -0.20  (-0.36, 0.06) | -0.12  (-0.20, -0.04) |

*Table S7: The proportion of the phenotypic correlation explained by bivariate additive genetic variance (A) and bivariate non-shared environmental variance (E) for positive life events and each of the wellbeing traits (95% confidence intervals in brackets).*

| Positive Life Events | | | |
| --- | --- | --- | --- |
|  | **Phenotypic Correlation** | **Proportion A** | **Proportion E** |
| Subjective Happiness | 0.12 (0.10, 0.15) | 0.42 (0.00, 0.79) | 0.43 (0.32, 0.45) |
| Life Satisfaction | 0.04 (0.01, 0.07) | 0.33 (0.00, 0.42) | 0.46 (0.29, 0.68) |
| Subjective Health | 0.09 (0.04, 0.13) | 0.26 (0.00, 1.00) | 0.55 (0.00, 0.81) |
| Hopefulness | 0.17 (0.13, 0.22) | 0.58 (0.20, 0.77) | 0.34 (0.14, 0.64) |
| Gratitude | 0.14 (0.09, 0.18) | 0.05 (0.00, 0.27) | 0.49 (0.27, 0.73) |
| Curiosity | 0.15 (0.11, 0.20) | 0.59 (0.06, 0.87) | 0.31 (0.06, 0.35) |
| Grit | 0.15 (0.11, 0.20) | 0.70 (0.39, 1.00) | 0.23 (0.00, 0.51) |
| Ambition | 0.19 (0.14, 0.23) | 0.50 (0.31, 0.79) | 0.44 (0.31, 0.61) |
| Optimism | 0.12 (0.07, 0.17) | 0.10 (0.00, 0.23) | 0.50 (0.17, 0.72) |
| Relatedness | 0.16 (0.12, 0.19) | 0.40 (0.08, 0.84) | 0.35 (0.19, 0.56) |
| Autonomy | 0.09 (0.06, 0.13) | 0.36 (0.00, 0.93) | 0.41 (0.15, 0.44) |
| Competence | 0.22 (0.18, 0.25) | 0.48 (0.18, 0.77) | 0.41 (0.27, 0.55) |
| Meaning in Life | 0.23 (0.20, 0.26) | 0.46 (0.17, 0.69) | 0.43 (0.39, 0.44) |
| Trust | 0.06 (0.03, 0.10) | 0.66 (0.56, 0.96) | 0.07 (0.00, 0.28) |

*Note: Proportions for gratitude and trust were calculated using absolute values because the correlations had different signs.*

*Table S8: The proportion of the phenotypic correlation explained by bivariate additive genetic variance (A) and bivariate non-shared environmental variance (E) for negative life events and each of the wellbeing traits (95% confidence intervals in brackets).*

| Negative Life Events | |  | |  | |
| --- | --- | --- | --- | --- | --- |
|  | **Phenotypic Correlation** | | **Proportion A** | | **Proportion E** |
| Subjective Happiness | -0.08 (-0.11, -0.06) | | 0.49 (0.04, 0.74) | | 0.51 (0.28, 0.71) |
| Life Satisfaction | -0.13 (-0.16, -0.10) | | 0.89 (0.46, 1.00) | | 0.09 (0.02, 0.19) |
| Subjective Health | -0.12 (-0.16, -0.07) | | 0.37 (0.00, 0.39) | | 0.42 (0.34, 0.73) |
| Hopefulness | -0.06 (-0.10, -0.01) | | 0.52 (0.00, 0.94) | | 0.44 (0.42, 0.79) |
| Gratitude | -0.08 (-0.13, -0.04) | | 0.09 (0.00, 0.76) | | 0.58 (0.28, 1.00) |
| Curiosity | 0.03 (-0.02, 0.08) | | - | | - |
| Grit | -0.08 (-0.13, -0.04) | | 0.74 (0.51, 1.00) | | 0.24 (0.00, 0.62) |
| Ambition | -0.07 (-0.11, -0.02) | | 0.57 (0.06, 0.95) | | 0.43 (0.42, 0.78) |
| Optimism | -0.08 (-0.12, -0.03) | | 0.55 (0.40, 0.88) | | 0.31 (0.26, 0.40) |
| Relatedness | -0.06 (-0.09, -0.02) | | 0.70 (0.20, 1.00) | | 0.18 (0.13, 0.25) |
| Autonomy | -0.10 (-0.13, -0.07) | | 0.51 (0.04, 0.82) | | 0.45 (0.18, 0.74) |
| Competence | -0.14 (-0.17, -0.11) | | 0.56 (0.36, 0.59) | | 0.45 (0.41, 0.64) |
| Meaning in Life | -0.08 (-0.11, -0.05) | | 0.36 (0.00, 0.60) | | 0.64 (0.50, 0.91) |
| Trust | -0.16 (-0.19, -0.13) | | 0.44 (0.00, 0.72) | | 0.44 (0.14, 0.73) |

**References**

1. Lyubomirsky S, Lepper HS (1999) A measure of subjective happiness: Preliminary reliability and construct validation. Soc Indic Res 46:137–155.

2. Huebner ES (1994) Preliminary development and validation of a multidimensional life satisfaction scale for children. Psychol Assess 6:149–158.

3. Seligson JL, Huebner ES, Valois RF (2003) Preliminary validation of the brief multidimensional students’ life satisfaction scale (BMSLSS). Soc Indic Res 61:121–145.

4. Morgan J, Farsides T (2009) Measuring meaning in life. J Happiness Stud 10:197–214.

5. Ryan RM, Deci EL (2000) The darker and brighter sides of human existence: Basic psychological needs as a unifying concept. Psychol Inq 11:319–338.

6. Gallup World Poll (2016) Gallup World Poll. In: Gallup.com. http://www.gallup.com/services/170945/world-poll.aspx. Accessed 31 Mar 2016

7. Snyder CR, Hoza B, Pelham WE, Rapoff M, Ware L, Danovsky M, Highberger L, Ribinstein H, Stahl KJ (1997) The development and validation of the Children’s Hope Scale. J Pediatr Psychol 22:399–421.

8. McCullough ME, Emmons RA, Tsang J-A (2002) The grateful disposition: a conceptual and empirical topography. J Pers Soc Psychol 82:112–127.

9. Scheier MF, Carver CS, Bridges MW (1994) Distinguishing optimism from neuroticism (and trait anxiety, self-mastery, and self-esteem): a reevaluation of the Life Orientation Test. J Pers Soc Psychol 67:1063–1078.

10. Duckworth AL, Peterson C, Matthews MD, Kelly DR (2007) Grit: perseverance and passion for long-term goals. J Pers Soc Psychol 92:1087.

11. Kashdan TB, Rose P, Fincham FD (2004) Curiosity and exploration: Facilitating positive subjective experiences and personal growth opportunities. J Pers Assess 82:291–305.

12. Duckworth AL, Quinn PD (2009) Development and validation of the Short Grit Scale (GRIT–S). J Pers Assess 91:166–174.

13. Ravens-Sieberer U, Gosch A, Rajmil L, Erhart M, Bruil J, Power M, Duer W, Auquier P, Cloetta B, Czemy L, others (2008) The KIDSCREEN-52 Quality of Life Measure for Children and Adolescents: Psychometric Results from a Cross-Cultural Survey in 13 European Countries. Value Health 11:645–658.
